# Cdc13 (cyclin B) is degraded by autophagy under sulfur depletion in fission yeast

**DOI:** 10.1080/27694127.2022.2047442

**Published:** 2022-03-22

**Authors:** Hokuto Ohtsuka, Yoshiko Hatta, Kana Hayashi, Takafumi Shimasaki, Yoko Otsubo, Yurika Ito, Yu Tsutsui, Nobutake Hattori, Akira Yamashita, Hiroshi Murakami, Hirofumi Aiba

**Affiliations:** 1 Laboratory of Molecular Microbiology, Graduate School of Pharmaceutical Sciences, Nagoya University; Nagoya, Aichi, Japan; 2 Laboratory of Cell Responses, National Institute for Basic Biology; Okazaki, Aichi, Japan; 3 National Institute for Fusion Science; Toki, Gifu, Japan; 4 Center for Novel Science Initiatives, National Institutes of Natural Sciences; Okazaki, Aichi, Japan; 5 Department of Basic Biology, School of Life Science, SOKENDAI (The Graduate University for Advanced Studies); Okazaki, Aichi, Japan; 6 Department of Biological Science, Faculty of Science and Engineering, Chuo University; Tokyo, Japan

**Keywords:** Autophagy, cyclin, Ecl1-family gene, fission yeast, TORC1

## Abstract

Cyclins are degraded by the anaphase-promoting complex/cyclosome (APC/C)-mediated proteasome in normal mitosis. We showed that Cdc13 (cyclin B) is also degraded by macroautophagy/autophagy in sulfur-deficient fission yeast. Sulfur depletion causes G_2_ cell cycle arrest and reduces cell size; however, the associated mechanisms are unknown. We found that autophagy is required for the degradation of Cdc13, which is associated with cell cycle arrest and reduced cell size, by examining cell morphology under sulfur depletion. The analysis of the Cdc13-GFP fusion protein supported the conclusion that Cdc13 is degraded by autophagy. Moreover, we showed that sulfur depletion results in the inactivation of target of rapamycin complex 1 (TORC1) activity via Ecl1-family proteins. Our data indicate that the cyclin is degraded by two different systems: APC/C-mediated proteasome and autophagy. The latter is induced under nutrient-depleted situations. This switch in degradation systems will contribute to appropriate cell cycle arrest when resources are depleted.

**Abbreviations:** APC, anaphase-promoting complex; CDK, cyclin-dependent kinase; DB, destruction box; EMM, Edinburgh minimal medium; GFP, green fluorescent protein; PCR, polymerase chain reaction; TOR, target of rapamycin; UPS, ubiquitin-proteasome system

## Introduction

Cell cycle regulation is governed by cyclin-dependent kinase (CDK) complex comprising cyclin and CDK [1–3]. The fission yeast, *Schizosaccharomyces pombe*, encodes six CDKs, Cdc2 being the essential one [4,5]. Several cyclins bind to and activate Cdc2 during mitosis, but only Cdc13 is essential [4]. The activity of Cdc2 is the lowest in G_1_, moderate in S, continues to rise through G_2_, and is the highest at the onset of mitosis [1]. Cdc13 is ubiquitinated by APC/C during mitosis, which acts as an E3 ubiquitin ligase, and degraded by the ubiquitin-proteasome system (UPS) [1,6–8]. Both the UPS and autophagy are the known proteolytic systems in eukaryotes [9]; however, cyclin degradation is thought to be associated only with UPS.

*S. pombe* enters the quiescent G_0_ stage from G_1_ and G_2_ [10,11]. Sulfur depletion leads to cell size reduction and cell cycle arrest at G_2_, extending the chronological lifespan (CLS) [12,13]. CLS extension via sulfur depletion depends on Ecl1-family genes. Ecl1-family genes are conserved in fungi, and *S. pombe* carries three Ecl genes (*ecl1*^+^, *ecl2*^+^, and *ecl3*^+^), which are induced by various types of nutrient depletion and stress, including sulfur depletion, amino acid depletion, nitrogen depletion, and magnesium depletion [14–17]. Sulfur depletion induces *ecl1*^+^, and then Ecl1 induces the expression of several autophagy-related (*Atg*) genes, suppresses expression of ribosomal protein genes, and contributes to the induction of autophagy and maintenance of cell viability [12,14,18]. Similarly, the target of rapamycin (TOR) pathway, which is conserved in mammals from yeast, also senses nutrient signals, such as nitrogen availability, and influences cellular processes, such as translational regulation, autophagy, and cellular lifespan [19–24]. *S. pombe* TOR complex 1 (TORC1) containing Tor2 kinase regulates various cellular processes via its targets including Psk1/S6 kinase and Atg13 (one of the Atg proteins) [20,25–27]. Both Ecl1-family genes and TORC1 respond to various trophic signals in *S. pombe* and induce environmental adaptation; however, the interrelationships among these proteins have not been clarified. Additionally, detailed cellular responses to sulfur depletion remain unknown.

## Results and Discussion

**Figure 1.**
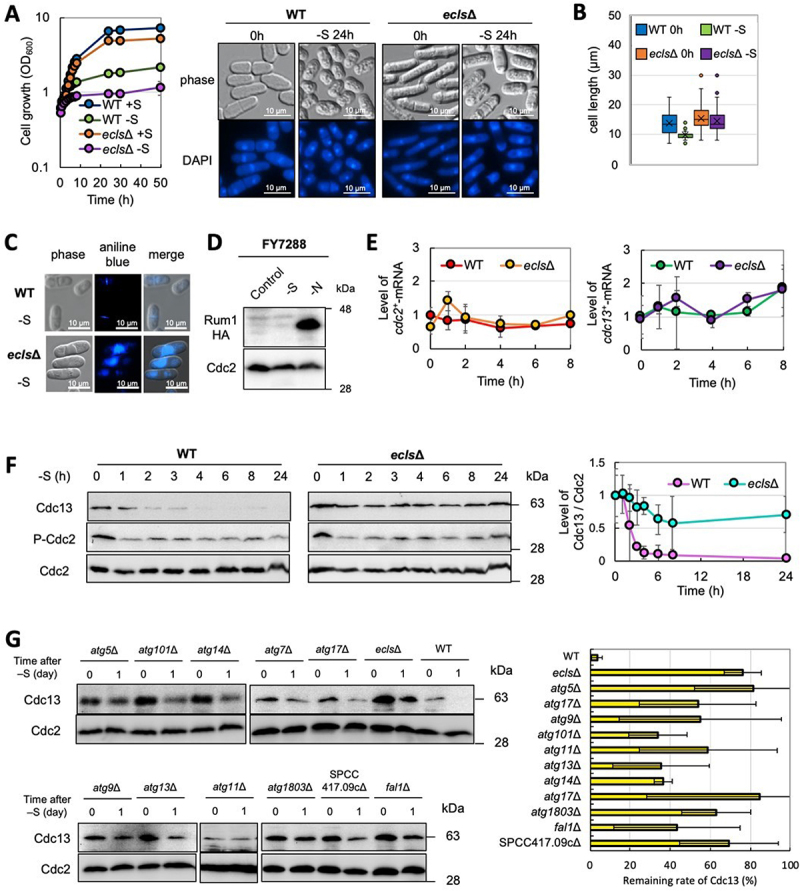
Destruction of cyclin Cdc13 requires Ecl1-family genes under sulfur depletion. (**A**) Cell growth of WT (JY333) and *ecls∆* (JY333 *ecl1∆ ecl2∆ ecl3∆*) cells after sulfur depletion (n = 3) (left panel). Cell morphology and 4’,6-diamidino-2-phenylindole (DAPI) staining of WT (JY333) and *ecls∆* (JY333 *ecl1∆ ecl2∆ ecl3∆*) cells (right panel). (**B**) WT (JY333) and *ecls∆* (JY333 *ecl1∆ ecl2∆ ecl3∆*) cells were cultured in Edinburgh minimal medium (EMM) to OD_600_ = 0.5 (0 h) and then transferred into sulfur-depleted EMM for 1 day (-S). The cell lengths were measured (n > 300). (**C**) Aniline blue staining of *ecls∆* cells during sulfur depletion. (**D**) FY7288 (Rum1-HA) cells were cultured in EMM to OD_600_ = 0.5 (control) and then transferred into sulfur-depleted EMM for 1 day (-S) and nitrogen-depleted EMM for 1 day (-N). (**E**) The expression of *cdc13*^+^ in WT (JY333) and *ecls∆* (JY333 *ecl1∆ ecl2∆ ecl3∆*) cells during sulfur depletion (n = 3). (**F**) Levels of Cdc13 and the Cdc2-Tyr15 phosphorylation under sulfur depletion (n = 3). **(G**) Cells were collected 1 day after transfer to sulfur-depleted medium, and levels of Cdc13 were analyzed (n = 3).

Sulfur depletion results in Ecl1-family gene-dependent reduction of the cell size of *S. pombe* [12] (Fig. 1A and 1B). *ecl1∆ ecl2∆ ecl3∆* (*ecls∆*) cells do not exhibit reduced cell size and develop abnormal deposits stained with aniline blue that stains the septum [28,29] (Fig. 1C). In addition, unlike nitrogen depletion, which is arrested in G_1_, sulfur depletion leads to G_2_ arrest [12]. In nitrogen depletion, CDK inhibitor Rum1 exists in a stable form, the CDK complex activity is sufficiently suppressed, and cells do not progress to S phase and halt at G_1_ [30–32]. In contrast, in sulfur depletion, Rum1 is not sufficiently present; hence, CDK retains some activity, and it is considered that cells progress to S phase, but not M phase, and halt at G_2_ (Fig. 1D).

To understand cellular responses to sulfur depletion, we used *S. pombe* deletion mutant library and comprehensively searched for mutants, which do not show cell size reduction under sulfur depletion. A total of 200 single-gene deletion strains were identified by this screening (Table S1).

To analyze epistasis between the identified genes and Ecl1-family genes, we introduced a plasmid expressing *ecl1*^+^ (pEcl1) in all 200 mutants and observed cell morphology under sulfur depletion. Ecl1 overexpression recovered cell morphology in 170 single-gene deletion mutants, suggesting these 170 genes act upstream of Ecl1-family gene. On the other hand, such recovery was not seen in the remaining 30 single-gene deletion strains (Table S2). Therefore, the latter 30 genes likely act downstream of Ecl1-family genes or contribute to cell size reduction by pathways independent of Ecl1-family genes.

Abnormal cell cycle progression in *S. pombe* is reflected in altered cell size distribution [32–34]. *ecls∆* cells do not normally respond to sulfur depletion, suggesting some dysfunction in cell cycle machinery. We thus measured three parameters in *ecls∆* cells under sulfur depletion: (i) mRNA levels of *cdc2*^+^ and *cdc13*^+^, (ii) degree of inhibitory phosphorylation of Tyr15 in Cdc2 [35], and (iii) protein levels of Cdc13 (Fig. 1E and 1F). Cdc13 levels declined in wild-type cells in response to sulfur depletion, but not in *ecls∆* cells (Fig. 1F), suggesting that the abnormal morphology of *ecls∆* cells during sulfur depletion is attributable to the failure of normal cyclin degradation. In addition, although absences of Ecl1-family genes have little effect on growth [36] (Fig. 1A), this triple deletion mutant appears to increase the level of Cdc13 under vegetative growth.

Cell size reduction and Cdc13 degradation do not occur in *ecls∆* cells under sulfur depletion. The 30 genes were identified as candidates for the required functions of cell size reduction under sulfur deficiency and may act downstream of the Ecl1-family genes (Table S2). We investigated the levels of Cdc13 in these mutants under sulfur depletion, as with *ecls∆* cells, 11 single-gene deletion mutants that did not display proper degradation were identified (Fig. 1G). Interestingly, nine of these genes were *Atg* genes, suggesting that autophagy is required for Cdc13 degradation.

**Figure 2.**
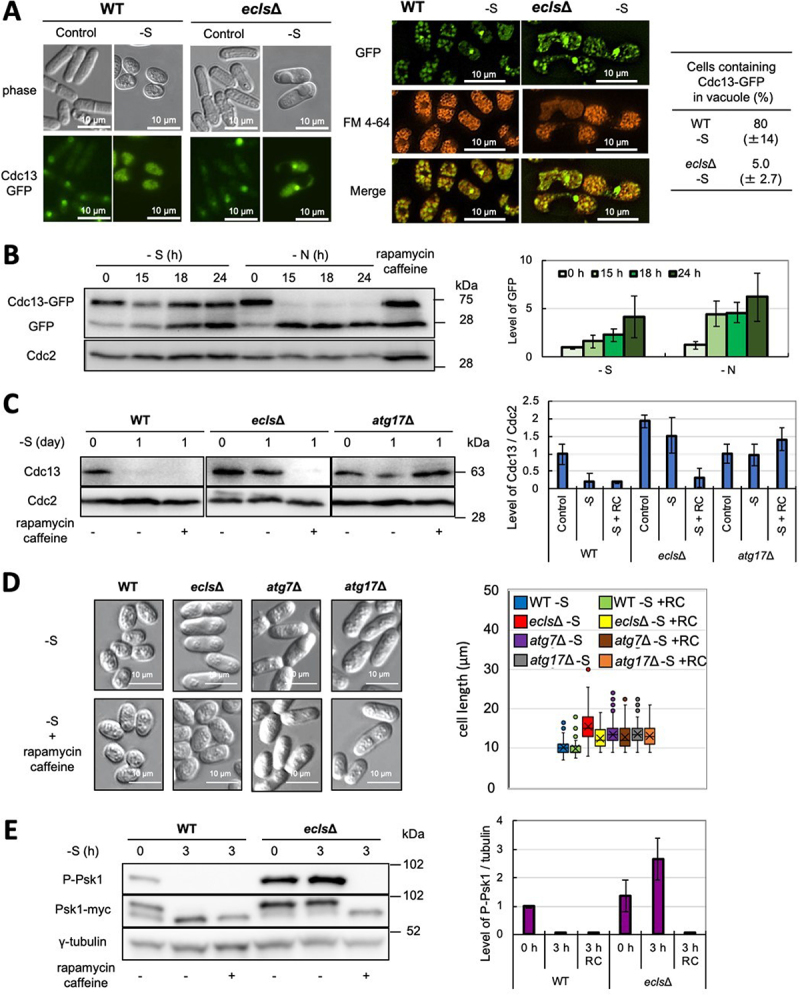
Autophagy degrades cyclin Cdc13 via Ecl1-family genes and TORC1 under sulfur depletion. (**A**) FY9210 (WT) and Cdc13GFP *ecl1∆ ecl2∆ ecl3∆* (*ecls∆*) cells were grown in Edinburgh minimal medium (EMM) to OD_600_ = 0.5 (control) and then transferred into sulfur-depleted EMM for 2 days (−S). (**B**) FY9210 cells were grown in EMM to OD_600_ = 0.5 and then transferred into sulfur-depleted EMM (−S) or nitrogen-depleted EMM (-N) (n = 3). (**C**) WT (JY333), *ecls∆* (JY333 *ecl1∆ ecl2∆ ecl3∆*), and a deletion set strain of *atg17∆* was grown in EMM to OD_600_ = 0.5 (0 day) and then transferred into sulfur-depleted EMM (1 day) (n = 3). (**D**) WT (JY333), *ecls∆* (JY333 *ecl1∆ ecl2∆ ecl3∆*), and deletion set strains of *atg7∆* and *atg17∆* were grown in EMM to OD_600_ = 0.5 (0 day) and then transferred into sulfur-depleted EMM (1 day). 9 mM caffeine and 200 ng/mL rapamycin were added at the same time as the transfer. The cell lengths were measured when rapamycin and caffeine were added (+RC) (n > 300). (**E**) WT (JY333 Psk1myc) and *ecls∆* (JY333 *ecl1∆ ecl2∆ ecl3∆* Psk1myc) cells were grown in EMM to OD_600_ = 0.5 (0 h) and then transferred into sulfur-depleted EMM (3 h) (n = 3).

In addition, analysis using Cdc13-GFP fusion proteins revealed that, in wild-type cells, almost Cdc13-GFP is localized in vacuoles, the destination of autophagy in yeast under sulfur depletion (Fig. 2A). Conversely, in *ecls∆* cells in which autophagy was not induced by sulfur depletion [18], proper localization of Cdc13-GFP into the vacuole was not observed. Meanwhile, its fluorescence intensity varied among the cells during logarithmic growth phase in both wild-type and *ecls∆* cells. This result is consistent with previous findings that Cdc13 is not consistently observed in mitotic cells [37], and Cdc13-GFP is likely to be periodically degraded by UPS in the presence of nutrients. From these findings, it was suggested that cyclin Cdc13 is transported to the vacuole by autophagy and degraded under sulfur depletion.

The state of degradation of Cdc13-GFP was also confirmed by western blot analysis (Fig. 2B). Accumulation of free-GFP, which is a degradation product of Cdc13-GFP, was observed in both sulfur- or nitrogen-starvation. It has been reported that the autophagy response due to sulfur starvation is delayed compared to that of nitrogen starvation [18,38].

Cdc13 degradation occurs by autophagy during periods of sulfur deficiency in WT cells. However, *ecls∆* cells do not degrade Cdc13 and do not display reduced cell size in similar conditions. Treatment with rapamycin and caffeine induces autophagy in *S. pombe* [39]. Under sulfur depletion, these drug treatments promote degradation of Cdc13 in *ecls∆* cells (Fig. 2C), thus suppressing the defect in Cdc13 degradation. Conversely, Cdc13 degradation does not occur in an *Atg* mutant, suggesting that degradation of Cdc13 under this condition is attributable to autophagy. Furthermore, drug treatment also ameliorates cell morphology in *ecls∆* cells (Fig. 2D), suggesting that the degradation of Cdc13 promotes the cell miniaturization even in this mutant. On the other hand, drug treatment did not lead to a reduction of cell size in an *Atg* mutant. Therefore, Cdc13 is degraded by autophagy in sulfur deficiency and this degradation may be important for miniaturization and proper G_0_-arrest of cells in a starved environment. Similarly, it has been reported that Cdc13 is stably present in the mutants in which cell miniaturization is not observed, such as *wis1-982* and *sty1-989* mutants, under nitrogen deficiency [40].

Rapamycin suppresses TORC1 activity [20]. Both Ecl1-family genes and TORC1 are involved in responses to nutrient depletion, but their relationship has not been investigated [14,20]. Phosphorylation of Psk1, a target of TORC1 [20], was examined using *ecls∆* cells to examine this relationship (Fig. 2E). Sulfur deficiency decreased the phosphorylation of the TORC1-phosphorylation site of Psk1. Conversely, in the absence of Ecl1-family genes, remarkable phosphorylation was observed with or without sulfur. Furthermore, the total level of Psk1 seems to be increased in this mutant. As shown in Fig. 2E, TORC1 activity of the *ecls∆* cells was much higher than that of control cells even in the logarithmic growth phase (0 hour), suggesting that Ecl1-family proteins also partially inhibit TORC1 activity in this minimum medium condition. Thus, Ecl1-family proteins have an important role to suppress the TORC1 activity, especially under conditions such as sulfur starvation.

**Figure 3.**
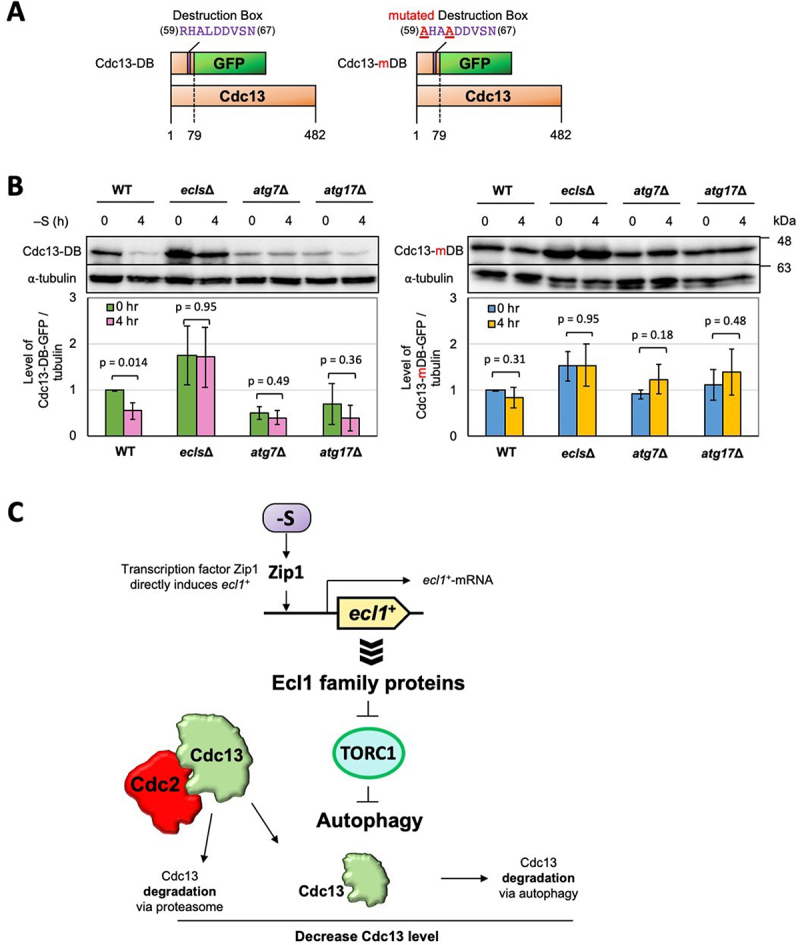
The destruction box of Cdc13 contributes to the autophagic degradation. (**A**) Structures of fusion proteins Cdc13-DB and Cdc13-mDB. (**B**) JY333 (WT) and *ecls∆* (JY333 *ecl1∆ ecl2∆ ecl3∆*) carrying pCdc13-DB or pCdc13-mDB plasmids were grown in EMM to OD_600_ = 0.5 (control) and then transferred into sulfur-depleted EMM for 4 h (n = 3). (**C)** A model illustrating intracellular responses during sulfur depletion in *S. pombe*. Sulfur starvation induces the expression of *ecl1*^+^ via Zip1 transcription factor [12]. Induced Ecl1-family proteins suppress the TORC1 activity and then lead the autophagy, which contributes to the degradation of Cdc13 during sulfur starvation.

In mitosis, Cdc13 is ubiquitinated by APC/C and degraded in the proteasome [41]. This ubiquitination is controlled by a highly conserved region, the destruction box (DB), at the N-terminus of the cyclin [6,42]. We created fusion proteins of GFP with the 79 amino acid N-terminal region of Cdc13 carrying DB or mutated DB (mDB) to investigate how this region participates in regulating autophagic degradation (Fig. 3A). Western blotting showed that DB proteins were degraded in WT cells but not in *ecls∆* and *Atg* deficient cells under sulfur depletion (Fig. 3B). This indicates that degradation of the fusion proteins during sulfur depletion depends on autophagy. On the other hand, mDB proteins were not degraded in these cells under sulfur depletion even in WT cells. This suggests that DB is required for not only degradation by UPS but also by autophagy. Meanwhile, in *Atg* mutants, the levels of Cdc13-DB-GFP but not Cdc13-mDB-GFP were lower than that of WT and *ecls∆* at 0 h (Fig. 3B). Several examples of cross-talk between autophagy and UPS have been reported [9]. Since these mutants do not have autophagy activity, UPS activity might be constitutively high, which results in the lower levels of Cdc13-DB-GFP in autophagy mutants.

Cdc13 is degraded during sulfur depletion, but the levels of its binding partner Cdc2 did not change (Fig. 1F and 1G), suggesting autophagy selectively degrades Cdc13 but not Cdc2 in this condition. Autophagy is a conserved proteolytic pathway from yeast to mammals; many Atg proteins have been identified in studies using *S. cerevisiae*, and similar proteins have been confirmed in *S. pombe* [43,44]. Atg11 is required for selective autophagy in *S. cerevisiae* but for general autophagy in *S. pombe* [45]. Atg11 but not Atg13, Atg17, or Atg101 in *S. pombe* is required for the kinase activity of Atg1 [46], suggesting that Atg protein functions may differ slightly among yeast species. At present, there is not much knowledge of the mechanisms of selective autophagy, especially that of ubiquitinated proteins, in *S. pombe* although three selective cargo receptors, Nbr1, Epr1, and Atg43 were reported [46–49]. Meanwhile, in other organisms, ubiquitinated proteins are selectively degraded by autophagy via autophagy receptors [9,50,51]. The DB of Cdc13 acts in cyclin ubiquitination [6] and is required for autophagy degradation under sulfur depletion (Fig. 3B). The selective degradation of Cdc13 during sulfur depletion may involve DB-mediated ubiquitination. This is one of the issues that needs to be analyzed in the future.

Sulfur depletion leads to a reduction in the cell size in *S. pombe*. Ecl1-family proteins appear to induce autophagy by repressing TORC1 (Fig. 3C). Cdc13, is degraded by autophagy, and the cell cycle is arrested in G_2_ and then enters the quiescent G_0_ state [12]. We found that the degradation of cyclin Cdc13 in *S. pombe* is due to autophagy in addition to UPS, suggesting that these two degradation processes act cooperatively to degrade and control Cdc13 (Fig. 3C). The accurate and rapid degradation of cyclin is required during the nutrient-rich logarithmic growth phase, and UPS degradation of cyclin is suitable for rapid cell proliferation. However, degradation by UPS requires substantial energy because it involves assembly of the proteasome complex, activation of ubiquitin, and unfolding of the protein substrate [9]. Conversely, the energy cost of autophagy is unclear; however, it is expected to be less than UPS because it is induced by starvation [9]. Autophagic degradation of cyclin during nutrient starvation may help control the cell cycle at a low energy cost when resources are scarce and maintain cell viability until resources in the environment are restored.

## Materials and Methods

### Strains and growth media

*Schizosaccharomyces pombe* strains are listed in Table S3. The *S. pombe* deletion mutant library from Bioneer (http://us.bioneer.com/products/spombe/spombeoverview.aspx) was used. Cells were grown in YE medium (0.5% yeast extract and 3% glucose) or Edinburgh minimal medium (EMM) [52] supplemented with essential nutrients as follows: 40 µg/mL adenine and 60 µg/mL leucine. Cells were grown at 30°C unless otherwise stated.

### Plasmid construction

The plasmid carrying *ecl1*^+^ (pEcl1), p825 [12], and the control plasmid, pLB-Dblet (vector) [12], were used. Plasmids, Cdc13-DB and Cdc13-mDB, were constructed by cloning the corresponding region in Fig. 3A onto plasmid pREP41-EGFP-C [53]. Primers used for cloning are listed in Table S3.

## *Constructions of the Cdc13GFP* ecl1*∆* ecl2*∆* ecl3*∆.*

The Cdc13-GFP *ecl1∆ ecl2∆ ecl3∆* strain was generated by mating cells of the FY9210 strain from the National Bio-Resource Project with cells of the JY808 *ecl1∆ ecl2∆ ecl3∆* strain [36].

## *Constructions of the JY333 Psk1myc and JY333* ecl1*∆* ecl2*∆* ecl3*∆ Psk1myc.*

For the detection of Psk1 protein, we fused a 13MYC-tag to the C terminus of Psk1 protein on chromosomes of JY333 and JY333 *ecl1∆ ecl2∆ ecl3∆* as previously described [54]. The primers used are listed in Table S3.

### Real-time polymerase chain reaction (PCR) analysis

Real-time PCR analysis was performed as previously described [55] using housekeeping gene *act1*^+^ as a control. The primers are listed in Table S3.

### Western blotting analysis

Western blotting was performed as previously described [18,20]. Anti-Cdc13 antibody (6F11/2; abcam, ab10873), anti-phospho-Cdc2 (Tyr15) antibody (Cell Signaling Technology, 9111), anti-Cdc2/CDK1 antibody (Y100.4; abcam, ab5467), monoclonal anti-Atb2/α-tubulin antibody (Sigma, T6074), anti-GFP (Roche, 11814460), monoclonal anti-Gtb1/γ-tubulin antibody (Sigma, T5326), MYC/c-Myc antibody (Santa Cruz Biotechnology, sc-40), and anti-phospho-Psk1/RPS6KB1/p70 S6 kinase (Thr389) (Cell Signaling Technology, 9206) were used.

### Microscopy observation

Microscopy observation was performed using the Nikon ECLIPSE Ti microscope. For DAPI staining, cells fixed with ethanol were stained with DAPI (Sigma, D9542) and observed. Cells were stained with aniline blue (Muto Pure Chemicals, 40202) for septal observation [28] and with FM 4-64 (Fujifilm, 222-02121) for vacuole observation [18]. In an experiment using FY9210 cells carrying Cdc13-GFP, compared to the findings for wild-type Cdc13, the cells exhibited slow miniaturization under sulfur depletion. Therefore, in this experiment (Fig. 2A), sulfur depletion was maintained for 2 days instead of 1 day.

### Statistical analysis

Quantitative data shown in figures represent the average of at least three independent experiments ±SD. Statistical analyses were done with the Student’s *t-test*.

## Supplementary Material

Supplemental Material

## Data and materials availability

All data are available in the main text or the supplementary materials.
